# Supplementary material for: Complexome profiling of the Chlamydomonas psb28 mutant reveals TEF5 as an early PSII assembly factor
Source: Plant Cell. 2025 Mar 17;37(6):koaf055. doi: 10.1093/plcell/koaf055 (PMC12164590; doi:10.1093/plcell/koaf055)
Supplement: koaf055_Supplementary_Data [file koaf055_supplementary_data.zip › psb28_interactive.zip!/plots/intensity/qualityPlots/peptides/SDH4_Cre01.g020305.t1.1.html]

### Cre01.g020305.t1.1

Trivial name: SDH4  
SeqCoverage [median]: 48.30  
Mass: 6.28 kDa  
  
 

| Sequence | MissedCleavage | Mass [Da] | wtIntensity | muIntensity | Score |
| --- | --- | --- | --- | --- | --- |
| MNLAGPGLTETVK | 0 | 1329.70 | 10411.07 | 22575.40 | 133.89 |
| VGVLGMSVVTYLGIMK | 0 | 1665.92 | 545.23 | 0.00 | 121.68 |
|  |  |  | 10956.3 | 22575.4 |  |
